# Efficacy analysis of different FLT3 inhibitors in patients with relapsed/refractory acute myeloid leukemia and high‐risk myelodysplastic syndrome

**DOI:** 10.1002/jha2.616

**Published:** 2022-11-21

**Authors:** Mahesh Swaminathan, Mai M. Aly, Abdul Moiz Khan, Bayan Al Share, Vikram Dhillon, Enxhi Lalo, Harry Ramos, Katherine G. Akers, Seongho Kim, Suresh Balasubramanian

**Affiliations:** ^1^ Department of Medicine Roswell Park Comprehensive Cancer Center Buffalo New York USA; ^2^ Clinical Hematology Unit Internal Medicine Department Assiut University Hospital Assiut Egypt; ^3^ Department of Oncology Karmanos Cancer Institute Wayne State University Detroit Michigan USA; ^4^ Department of Internal Medicine Wayne State University School of Medicine Detroit Michigan USA; ^5^ Wayne State University School of Medicine Detroit Michigan USA; ^6^ Shiffman Medical Library Wayne State University Detroit Michigan USA; ^7^ Biostatistics and Bioinformatics Core Karmanos Cancer Institute Wayne State University Detroit Michigan USA; ^8^ Translational Hematology and Oncology Research Taussig Cancer Institute, Cleveland Clinic Cleveland Ohio USA

**Keywords:** FLT3 inhibitors, meta‐analysis, relapsed/refractory acute myeloid leukemia, type 1, type 2

## Abstract

Several FLT3 inhibitors(i) are available to treat relapsed/refractory (R/R) *FLT3*‐internal tandem duplicated acute myeloid leukemia (AML). This study analyzes the efficacies of various FLT3i (types 1 and 2) tested in clinical trials in treating R/R AML and high‐risk myelodysplastic syndromes (HR‐MDS). PubMed and EMBASE databases were searched for single/double‐arm phase I/II/III R/R AML or HR‐MDS clinical trials published between 1/1/2000 and 6/1/2021. The outcomes studied were composite response rate (CRc) and overall response rate (ORR). Toxicities were compared based on the organ system. The 28 studies analyzed had 1927 patients. The pooled ORR and (CRc) for all FLT3i were 53% (95% CI, 43%–63%) and 34% (95% CI, 26%–44%). Pooled ORR and CRc were 37% (95% CI, 25%–51%) and 35% (95% CI, 21%–52%) for type 1 and 58% (95% CI, 43%–71%) and 38% (95% CI, 27%–50%) for type 2, respectively. Gastrointestinal (GI) and hematological toxicity occurred in 22% (95% CI, 19%–25.4%) and 74.6% (95% CI, 70%–79%) with type 1 and 13.9% (95% CI, 12%–16%) and 57.7% (95% CI, 54.6%–60.8%) with type 2 FLT3i. QTc prolongation occurred in 2.06% (95% CI, 1.03%–3.65%) with type 1 and 7% (95% CI, 5.3%–9%) with type 2 FLT3i. Type 2 FLT3i had less GI toxicity but more QTc prolongation. Prospective studies are needed to compare the efficacy of type 1 and 2 FLT3i.

## 1 INTRODUCTION

Next‐generation sequencing has revealed the molecular landscape and complex clonal evolution in acute myeloid leukemia (AML) and high‐risk myelodysplastic syndromes (HR‐MDS) [1]. Defining the molecular background of AML identified recurrent somatic events implicated in leukemogenesis and led to targeted treatment strategies and improved outcomes [2]. Mutations in the FMS‐like tyrosine kinase 3 (*FLT3*) gene are the most frequent somatic events in newly diagnosed AML patients [3]. *FLT3* mutations either occur as the more common (∼30% of patients of de novo AML) internal tandem duplication (ITD), an in‐frame duplication, or the less frequent (∼5%–10% of de novo AML) tyrosine kinase domain (TKD) point mutation [4, 5, 6, 7]. *FLT3*‐ITD mutations confer adverse prognosis and are considered a driver lesion, especially at a higher allelic ratio [3, 8–10]. Approximately more than half of *FLT3*‐mutated de novo AML patients tend to harbor the *FLT3* mutation at relapse [11]; however, the number at relapse is predicted to be less in the midostaurin era. The substantial presence of *FLT3* mutation at relapse suggests that the *FLT3‐*ITD clone present at the initial diagnosis can undergo clonal expansion resulting in relapsed AML [11]. Considering the prevalence and poor prognosis of *FLT3‐*ITD mutated AML, targeting the *FLT3* signaling pathway became a promising therapeutic approach.

There are two generations of FLT3 inhibitors currently in clinical practice: first‐ (e.g., sorafenib, midostaurin) and second‐generation FLT3 inhibitors (e.g., gilteritinib, quizartinib). First‐generation FLT3 inhibitors are multi‐kinase nonspecific inhibitors, whereas the second generation is more specific and potent FLT3 inhibitors [7]. FLT3 inhibitors are further divided into type 1 (e.g., midostaurin, gilteritinib) versus type 2 (e.g., sorafenib, quizartinib) depending on how they interact with the intracellular kinase domain of the FLT3 receptor (Figure S1). Type 1 binds to the FLT3 receptor in both active and inactive conformation and is active against both *FLT3*‐ITD and ‐TKD mutations. In comparison, type 2 binds to a hydrophobic region adjacent to the ATP‐binding pocket in the inactive conformation and targets only FLT3‐ITD mutation [7]. Currently, gilteritinib is the only FLT3 inhibitor approved by the Food and Drug Administration (FDA) for treating patients with *FLT3*‐mutated relapsed/refractory (R/R) AML. Although several FLT3 inhibitors have been studied to treat FLT3‐mutated R/R AML patients [12], there are no direct prospective comparative studies to analyze these different FLT3 inhibitors. Although the extant classification is based on the pharmacological properties and mechanism of action of the drugs, its usefulness in understanding their efficacy and toxicity profile is unclear.

Therefore, we conducted this meta‐analysis wherein we analyzed the efficacy of FLT3 inhibitors as monotherapy in patients with R/R AML and HR‐MDS. We will discuss FLT3 inhibitors individually and elucidate the efficacy of type 1 and type 2 FLT3 inhibitors.

## 2 METHODS

Standard systematic review methods were used and reported according to the preferred reporting items for systematic reviews and meta‐analyses (PRISMA) guidelines. The protocol was registered on PROSPERO CRD42021267536. PubMed and EMBASE databases were searched for clinical trials published between 1/1/2000 and 6/1/2021 using keywords and subject headings related to FLT3 inhibitors and AML. Two independent reviewers screened titles/abstracts and full texts, with a third reviewer resolving conflicts. Studies were included if (1) full‐length published journal articles that (2) reported the results of single‐ or double‐arm phase I/II/III clinical trials in patients with R/R AML or HR‐MDS were available. Outcomes of interest were composite response rate (CRc = complete response [CR] + complete response with incomplete count recovery) and overall response rate (ORR).

The heterogeneity test was performed using Cochran's *Q* test and *I* ^2^ values. The presence of heterogeneity was considered if either Cochran's *Q* test *p*‐value was <0.10 or *I* ^2^ values ≥ 50%. If the heterogeneity tests were significant, a random‐effect model was used. Consequently, a random‐effect model was used to calculate pooled estimates for all outcomes (CRc and ORR). The publication bias was evaluated using a funnel plot and Egger's linear regression test. If significant, the trim‐and‐fill method was used to estimate and adjust for the number and outcomes of missing studies in a meta‐analysis. Egger's linear regression test showed that all outcomes have publication bias, and the trim‐and‐fill method was applied to estimate the adjusted pooled estimates.

## 3 RESULTS

**FIGURE 1:**
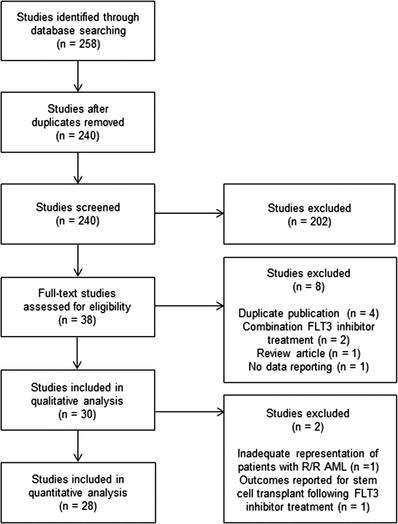
Preferred reporting items for systematic reviews and meta‐analyses (PRISMA) guidelines diagram. Database (EMBASE, PubMed) search identified 258 studies for screening. A total of 30 studies were included in the qualitative analysis. Two were excluded for quantitative analysis due to inadequate representation of the population of interest and outcomes assessed for intervention other than FLT3 inhibitors.

Database (EMBASE, PubMed) search identified 258 studies for screening. A total of 30 studies were included in the qualitative analysis (Figure 1). Two were excluded for quantitative analysis due to inadequate representation of the population of interest and outcomes assessed for intervention other than FLT3 inhibitors.

The study characteristics of all 28 studies involving 1927 patients are summarized in Table 1. The median age of patients included in this meta‐analysis was 62 years (range, 45–71 years). FLT3 inhibitors that were investigated in the included studies were crenolanib (one phase I/II study), EMND‐2076 (one phase I study), FLX925 (one phase I/II study), gilteritinib (one phase I, I/II, and III studies, respectively), lestaurtinib (one phase I/II study), linifanib (one phase I study), midostaurin (one phase I and phase II study, respectively), pacritinib (one phase I study), pexidartinib (one phase I/II study), ponatinib (one phase I study), quizartinib (one phase II study, three phase II studies, and one phase III study), semaxanib (two phase II studies), sorafenib (four phase I studies and one phase II study), sunitinib (one phase I study), and tandutinib (one phase I study).

**TABLE 1:** Baseline characteristic of 28 clinical trials included in the meta‐analysis

Author and year	Identifier	Drug	Study phase/study type	Patients (N)/(MDS)	Age median (range)	Gender (male)
**Galanis et al**. [13]	NCT01657682	Crenolanib^a^	I–II/open label study	65	61 (30–87)	N/A
**Yee et al**. [14]	N/A	EMND‐2076^b^	I/open label, randomized	16	69 (43–84)	20
**Daver et al**. [15]	NCT02335814	FLX925^a^	I–II/open label study	51	56 (18–71)	21
**Perl et al**. [16]	NCT02014558	Gilteritinib^a^	I–III/open‐label	249	63 (47–71)	129
**Usuki et al**. [17]	NCT02181660	Gilteritinib^a^	I/open label, randomized	19	70 (60–81)	15
**Perl et al**. [18]	NCT02421939	Gilteritinib^a^	III/randomized	247	61.5 (19–85)	116
**Smith et al**. [19]	N/A	Lestaurtinib^a^	I–II/open label single arm	17	56 (18–71)	9
**Wang et al**. [20]	N/A	Linifanib^b^	I/dose escalation	26	54 (24–81)	16
**Stone et al**. [21]	N/A	Midostaurin^a^	I/single arm	18 (1)	62 (29–78)	14
**Fischer et al**. [22]	NCT00045942	Midostaurin^a^	II/open label, randomized	67 (10)	62 (18–71)	49
**Jeon et al**. [23]	N/A	Pacritinib^a^	I/pilot	6	56 (33–76)	4
**Smith et al**. [24]	NCT01349049	Pexidartinib^a^	I–II/single arm open label	90	58 (22–83)	46
**Shah et al**. [25]	N/A	Ponatinib^b^	I/single arm	12	50 (30–72)	7
**Cortes et al**. [26]	NCT01565668	Quizartinib^b^	II/single‐arm	333	60 (39–73)	170
**Cortes et al**. [27]	NCT02039726	Quizartinib^b^	III/randomized	245	55 (46–65)	113
**Cortes et al**. [28]	NCT00462761	Quizartinib^b^	I/randomized	76	60 (23–86)	46
**Cortes et al**. [29]	NCT01565668	Quizartinib^b^	II/randomized	76	55 (19–77)	44
**Usuki et al**. [30]	NCT02675478	Quizartinib^b^	I/dose escalation	16	68 (33–91)	9
**Takahash et al**. [31]	NCT02984995	Quizartinib^b^	II/single‐arm	27	65 (31–81)	15
**Giles et al**. [32]	N/A	Semaxinib^b^	II/single‐arm	55 (22)	64 (23–76)	39
**Fiedler et al**. [33]	N/A	Semaxinib^b^	II/single arm	25	65 (27–79)	26
**Man et al**. [34]	N/A	Sorafenib^b^	II/open label single arm	13	45 (13–69)	2
**Borthakur et al**. [35]	NCT00217646	Sorafenib^b^	I/randomized	50 (1)	60 (21–88)	25
**Borthakur et al**. [36]	NCT00943943	Sorafenib^b^	I/single arm	28	58 (18–85)	12
**Pratz et al**. [37]	N/A	Sorafenib^b^	I/dose escalation	14	63 (37–50)	8
**Crump et al**. [38]	N/A	Sorafenib^b^	I/randomized	32 (4)	71 (37–82)	32
**Fiedler et al**. [39]	N/A	Sunitinib^a^	I/single arm	16	64 (55–80)	6
DeAngelo et al. [40]	MLN518/CT53518	Tandutinib^b^	I/single arm	40 (1)	70.5 (22–90)	28

^a^ Type 1 FLT3 inhibitor.

^b^ Type 2 FLT3 inhibitor.

Abbreviations: MDS, myelodysplastic syndromes; N/A, not available.

The efficacy and safety results are described in the following sections.

### 3.1 Efficacy analysis

#### 3.1.1 All FLT3 inhibitors combined (including type 1 and type 2)

**FIGURE 2:**
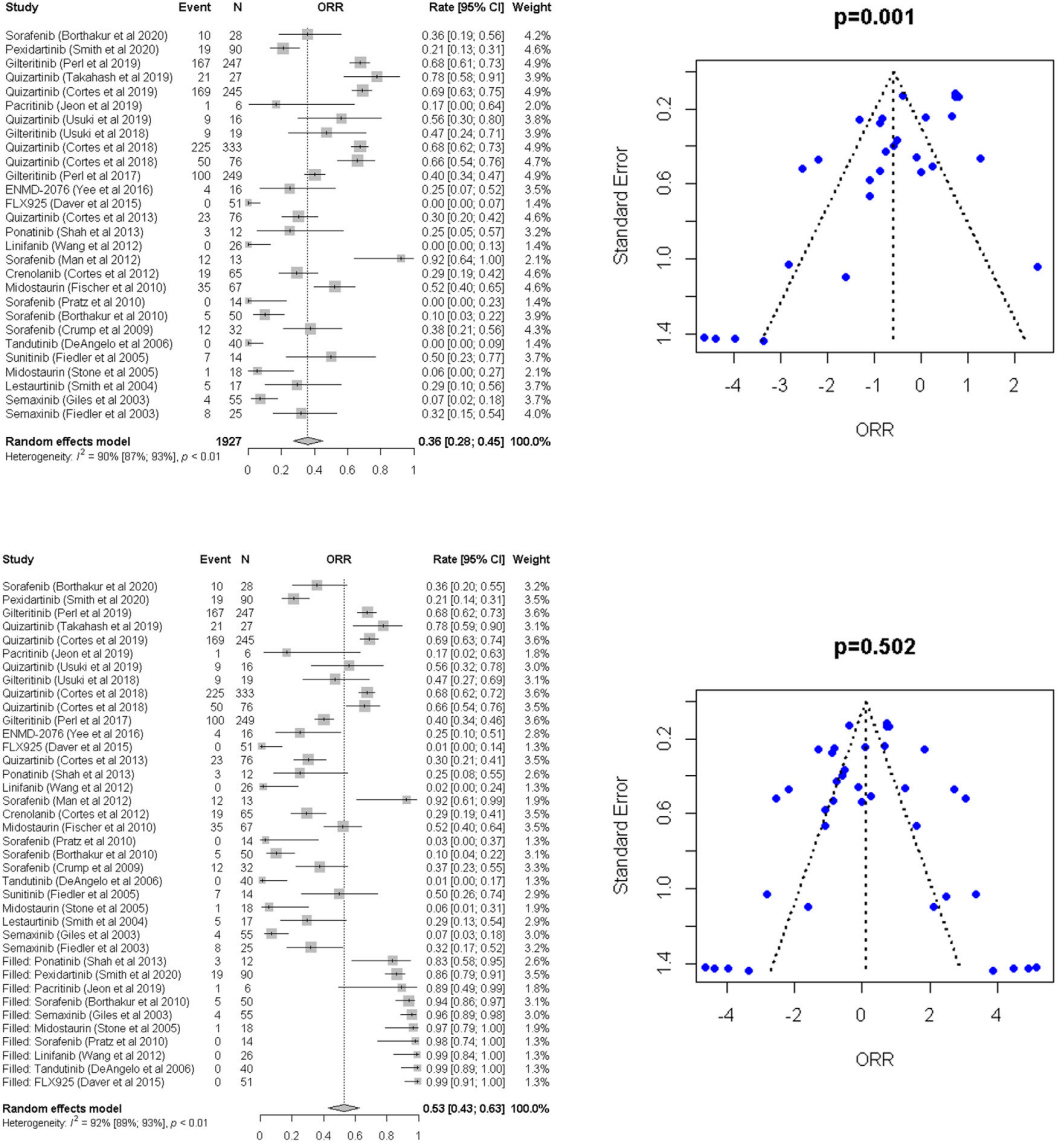
Top: Forest plot of the meta‐analysis and funnel plot for publication bias for overall response rate (ORR). In the funnel plot, the dotted line represents a triangular 95% confidence region and the pooled estimate based on a random‐effect meta‐analysis. Bottom: Forest plot of the meta‐analysis and funnel plot for publication bias for overall response rate (ORR) after adjusting publication bias by the trim‐and‐fill method. In the funnel plot, the dotted line represents a triangular 95% confidence region and the pooled estimate based on a random‐effect meta‐analysis.

The Cochran's *Q* test *p*‐value was less than 0.10 (*p* < 0.01), and *I* ^2^ value was more than 50% (*I* ^2^ = 87%), indicating the presence of heterogeneity (Figure 2, top‐left). Thus, random‐effects models were used. Asymmetry test performed using Egger's linear regression test (Figure 2, top‐right) suggested publication bias (*p* = 0.001; Figure 2, top‐right). The trim‐and‐fill method used to adjust publication bias showed the pooled ORRs to be 53% (95% CI, 43%–63%) (Figure 2, bottom).

**FIGURE 3:**
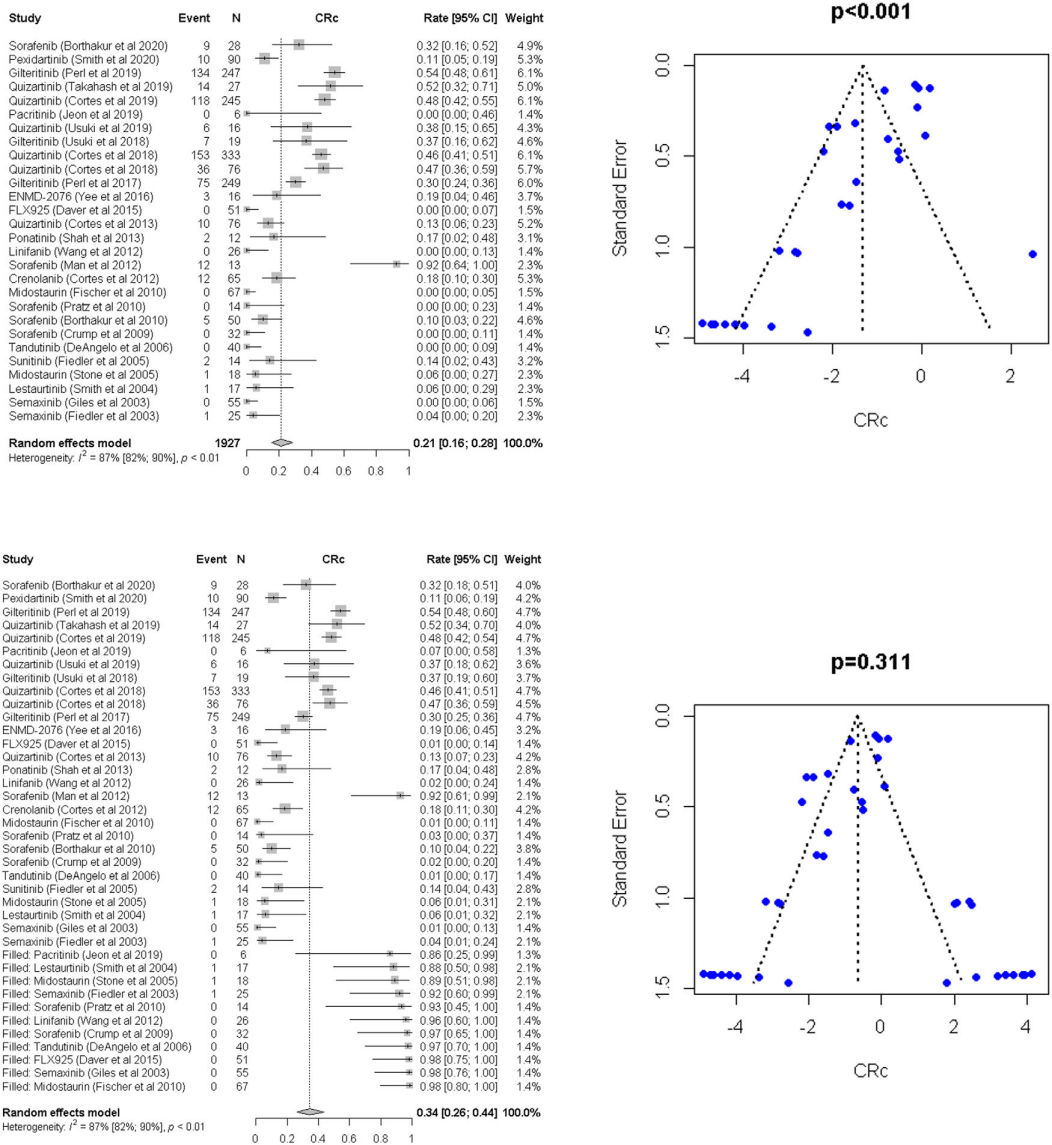
Top: Forest plot of the meta‐analysis and funnel plot for publication bias for overall response rate (ORR) after adjusting publication bias by the trim‐and‐fill method. In the funnel plot, the dotted line represents a triangular 95% confidence region and the pooled estimate based on a random‐effect meta‐analysis. Bottom: Forest plot of the meta‐analysis and funnel plot for publication bias for complete response rate (CRR) after adjusting publication bias by the trim‐and‐fill method. In the funnel plot, the dotted line represents a triangular 95% confidence region and the pooled estimate based on a random‐effect meta‐analysis.

The Cochran's *Q* test *p*‐value was less than 0.10 (*p* < 0.01), and *I* ^2^ value was more than 50% (*I* ^2^ = 87%), indicating the presence of heterogeneity (Figure 3, top‐left). Thus, random‐effects models were used. Asymmetry test performed using Egger's linear regression test (Figure 3, top‐right) suggested publication bias (*p* < 0.001; Figure 3, top‐right). The trim‐and‐fill method used to adjust publication bias showed the pooled composite response rates (CRc) was 34% (95% CI, 26%–44%) (Figure 3, bottom).

#### 3.1.2 Individual FLT3 inhibitors

The pooled estimates of ORR and CRc of individual FLT3 inhibitors are summarized in Table 2. The pooled response rates were estimated by random effect models when the number of studies was two or more. Quizartinib, sorafenib, and gilteritinib were the most frequently evaluated FLT3 inhibitors, with 6, 5, and 3 studies included in our analysis comprising 773, 137, and 515 patients, respectively.

**TABLE 2:** Summary of pooled estimates by type of medication

			Pooled rate (95% CI)^a^	
Drug	No. of studies	No. of patients	ORR	CRc
Quizartinib	6	773	0.61 (0.49,0.72)	0.40 (0.31,0.51)
Gilteritinib	3	515	0.52 (0.31,0.73)	0.40 (0.23,0.60)
Sorafenib	5	137	0.31 (0.12,0.60)	0.20 (0.04,0.56)
Pexidartinib	1	90	0.21 (0.14,0.31)	0.11 (0.06,0.19)
Midostaurin	2	85	0.23 (0.02,0.84)	0.03 (0.00,0.16)
Semaxinib	2	80	0.16 (0.03,0.53)	0.02 (0.00,0.11)
Crenolanib	1	65	0.29 (0.19,0.41)	0.18 (0.11,0.30)
FLX925	1	51	0.01 (0.00,0.14)	0.01 (0.00,0.14)
Tandutinib	1	40	0.01 (0.00,0.17)	0.01 (0.00,0.17)
Linifanib	1	26	0.02 (0.00,0.24)	0.02 (0.00,0.24)
Lestaurtinib	1	17	0.29 (0.13,0.54)	0.06 (0.01,0.32)
ENMD‐2076	1	16	0.25 (0.10,0.51)	0.19 (0.06,0.45)
Sunitinib	1	14	0.50 (0.26,0.74)	0.14 (0.04,0.43)
Ponatinib	1	12	0.25 (0.08,0.55)	0.17 (0.04,0.48)
Pacritinib	1	6	0.17 (0.02,0.63)	0.07 (0.00,0.58)

^a^ Pooled rates were estimated by random effects models when the number of studies is 2 or more.

Abbreviations: CI, confidence interval; CRc, complete response rate; ORR, overall response rate.

For quizartinib, sorafenib and gilteritinib, the pooled ORR were 61% (95% CI 49%–72%), 31% (95% CI 12%–60%) and 52% (95% CI 31%–73%), whereas the pooled composite response rates (CRc) were 40% (95% CI 31%–51%), 20% (95% CI 4%–56%), and 40% (95% CI 23%–60%), respectively.

#### 3.1.3 Type 1 versus type 2 FLT3 inhibitors

Cochran's *Q* test *p*‐values were less than 0.10 (*p* < 0.01 for both types 1 and 2), and *I* ^2^ values were more than 50% (*I* ^2^ = 88% for type 1 and 92% for type 2), indicating the presence of heterogeneity. Using Egger's linear regression test, asymmetry tests were performed, suggesting publication bias for type 2 (*p* = 0.005). After adjusting for publication bias for type 2 using the trim‐and‐fill method, the pooled ORR was calculated to be 37% (95% CI, 25%–51%) for type 1 and 58% (95% CI, 43%–71%) for type 2 (*p* = 0.258) FLT3 inhibitors. Similarly, pooled CRc was 35% (95% CI, 21%–52%) for type 1 and 38% (95% CI, 27%–50%) for type 2 (*p* = 0.460) FLT3 inhibitors (Figure S2).

Three prechosen categorical variables (number of prior lines, history of stem cell transplant, and prior use of FLT3 inhibitor) were evaluated for sources of heterogeneity using univariable meta‐regression models before and after correction of publication bias by a trim‐and‐fill method. Before the correction of publication bias, we observed that there were significant associations between the number of prior lines and ORR (*p* = 0.016), not for the history of stem cell transplant (*p* = 0.729) and the prior use of FLT3 inhibitor (*p* = 0.910), and between the history of stem cell transplant and CRc (*p* = 0.001), not for the number of prior lines (*p* = 0.374) and the prior use of FLT3 inhibitor (*p* = 0.327). However, after correction of the publication bias, ORR and CRc were not significantly associated with the number of prior lines, the history of stem cell transplant, and the prior use of FLT3 inhibitor (*p* = 0.743, 0.447, and 0.776 for ORR; *p* = 0.362, 0.801, and 0.558 for CRc, respectively).

### 3.2 Adverse events

Most common adverse events with all FLT3 inhibitors were gastrointestinal (GI) (16.9%; 95% CI, 15.2%–18.6%), hematologic (62.3%; 95% CI, 59.7%–64.9%), and cardiac toxicity, specifically QTc prolongation (4.9%; 95% CI, 3.8%–6.3%) (Table 3). GI toxicity occurred in 22.1% (95% CI, 19%–25.4%) with type 1 and 13.9% (95% CI, 12%–16%) with type 2 inhibitors. Hematologic toxicity was also higher with type 1 than type 2, as it occurred in 74.6% (95% CI, 70%–79%) with type 1 and 57.7% (95% CI, 54.6%–60.8%) with type 2, however; QTc prolongation occurred only in 2% (95% CI, 1%–3.6%) with type 1 and 7% (95% CI, 5.3%–9%) with type 2. The type 2 FLT3 inhibitors that were more associated with prolonged QTc were quizartinib (7.1%; 95% CI, 5.3%–9.2%) and ENMD‐2076 (3.7%; 95% CI, 0.1%–19%). Similarly, the type 1 inhibitors that caused QTc prolongation were gilteritinib (1.9%; 95% CI, 0.9%–3.5%) and pacritinib (7.7%; 95% CI, 0.20%–36%).

**TABLE 3:** Major adverse events with FLT3 inhibitors

Toxicity	All FLT3 inhibitors	Type 1 inhibitors	Type 2 inhibitors
**GI toxicity**	16.9% (95% CI, 15.2%–18.6%)	22.1% (95% CI, 19%–25.4%)	13.90% (95% CI, 12%–1%)
**Hematologic toxicity**	62.3% (95% CI, 59.72%–64.94%)	74.6% (95% CI, 70%–79.%)	57.7% (95% CI, 54.6%–60.8%)
**Cardiac toxicity (QTc prolongation)**	4.9% (95% CI, 3.8%–6.3%)	2% (95% CI, 1%–3.6%)	7% (95% CI, 5.3%–9%)

Abbreviation: GI, gastrointestinal.

## 4 DISCUSSION

*FLT3* mutation and preferential targeting in AML have changed the therapeutic armamentarium in the management of AML. Approximately half of the patients have persistence of *FLT3* mutated clone at the time of relapse, which makes targeting *FLT3* mutation of prime importance in patients with R/R AML. Although gilteritinib (type 1 FLT3 inhibitor) is the only FDA‐approved FLT3 inhibitor to treat patients with *FLT3*‐mutated R/R AML, a perusal of the literature showed no direct comparisons among FLT3 inhibitors in the treatment of patients with R/R AML. FLT3 inhibitors differ in their mechanism of action depending on their target site on the FLT3 receptor. Whether type 1 FLT3 inhibitors are more effective than type 2 because of relatively broader activity is still yet to be answered. This systematic review and meta‐analyses attempted to understand the differences among different FLT3 inhibitors in general and between type 1 and type 2 FLT3 inhibitors in treating R/R AML and HR‐MDS patients. With the availability of numerous FLT3 inhibitors, what may still be unanswered is the order of sequence in using type 1, and 2 FLT3 inhibitors in de novo and R/R *FLT3*ITD‐mutated AML.

FLT3 inhibitors were shown to be safe and effective during induction, re‐induction, and post‐allogeneic stem cell transplant in patients with untreated FLT3‐mutated AML in prior systematic reviews and meta‐analyses [41, 42]. However, there are no studies evaluating the safety and efficacy of FLT3 inhibitors as monotherapy in R/R AML. Historically, patients with *FLT3*‐mutated R/R AML have ORR and CRc rates of 26% and 22% with salvage chemotherapy, respectively [18]. In this study, the pooled ORR and CRc rates in patients treated with FLT3 inhibitors were 53% (95% CI, 43%–63%) and 34% (95% CI, 26%–44%), respectively. Although we acknowledge that most of the studies included in our meta‐analyses were single‐arm/open‐label trials and the limitations of cross‐trial comparisons, the pooled response rates observed in our study suggest that FLT3 inhibitors are quite effective in the treatment of patients with R/R AML who are likely predicted to be chemo‐refractory. Also, it is essential to note that some of the patients included in our meta‐analyses were heavily pre‐treated before their treatment with FLT3 inhibitor.

Type 1 and 2 FLT3 inhibitors differ in their target sites on the FLT3 receptor and mechanisms of resistance. Alotaibi et al. showed that the most common emergent mutations in patients treated with type 1 FLT3 inhibitors were in the RAS/MAPK pathway, whereas *FLT3*‐D835, *IDH1*/*IDH2*, and *TP53* were the common emergent escape mutations in patients treated with type 2 FLT3 inhibitors [43]. The latter suggests that type 1 and 2 FLT3 inhibitors have distinct biological implications, which may translate into different efficacies and toxicities. Although the current classification of FLT3 inhibitors based on the pharmacological properties is relevant in understanding their mechanisms of action, toxicity, and the differential emergent patterns of resistance, as well as drug selection based on *FLT3*‐ITD versus TKD mutation, translating this into clinical efficacy may not be as straightforward. There is significant heterogeneity in properties such as potency, selectivity, and protein binding within the types of FLT3 inhibitors; therefore, a knowledge of the individual drugs is still vital to appropriate drug selection and sequencing them for the patients.

Our analyses showed that the pooled response rates were not significantly different between type 1 and 2 inhibitors. However, there was a trend toward a higher pooled ORR in patients treated with type 2 FLT3 inhibitors (58% vs. 37%). When we look at the individual drugs, appreciating the caveat that cross‐trial comparisons are impractical to derive any definitive conclusions, the suggestion of a numerically higher ORR with type 2 inhibitors becomes less generalizable. Gilteritinib, a type I inhibitor, has a considerable ORR of 52% (95% CI 31%–75%), only behind 61% (95 CI 49%–72%) ORR of quizartinib, which is a type 2 inhibitor. A consistent pattern of efficacy based on the type of inhibitors also fails to emerge when looking at the other drugs, indicating that the numerically higher pooled ORR of type 2 inhibitors is likely driven by quizartinib by virtue of its largest sample size in our study (773 patients from 6 studies). This also underscores that the optimum choice of FLT3 inhibitor in de novo and R/R setting based on emergent resistance mutations and different biological implications can only be answered in a prospective study.

The toxicity profile was variable between type 1 and 2 FLT3 inhibitors. Hematological toxicities were more common in type 1 FLT3 inhibitors (74.6% vs. 57.7%), and QTc were more frequent in type 2 FLT3 inhibitors (7% vs. 2%). This is key information to note as there are ongoing studies evaluating the safety and combination of FLT3 inhibitors with the standard of care regimens such as venetoclax, hypomethylating agent (NCT04140487, NCT04687761), and intensive chemotherapy (NCT04047641, NCT02668653, NCT04027309).

Several limitations in our study should be mentioned. First, we did not include survival estimates in the outcomes assessment as some included studies were early‐phase clinical trials. Also, depending on the time periods of the included studies, the frontline treatment would have been different in our study patients, which would impact the outcomes at the time of relapse. For example, midostaurin was approved by the FDA only in 2017. Some studies included in our systematic review (18/28) were conducted before midostaurin approval. Second, our study did not include FLT3 inhibitors evaluated as combination therapies in patients with R/R AML. We excluded such studies as the current standard of care for patients with *FLT3*‐mutated R/R AML is gilteritinib monotherapy, and we wanted to deduce the actual efficacy of the FLT3 inhibitor per se. However, it is vital to note that the LACEWING trial, which showed no significant survival benefit of adding azacitidine to gilteritinib, had more than a modicum of benefit with the combination, especially CRc [44]. This highlights the importance of evaluating the efficacy of different FLT3 inhibitors in combination with chemotherapy in treating patients with *FLT3*‐mutated R/R AML.

In conclusion, our study showed there was a trend to a nonsignificantly higher ORR in patients treated with type 2 FLT3 inhibitors as monotherapy in the treatment of R/R AML, which is broadly an effect of a large sample size rather. However, this will still be an important yet unanswered question about the clinical difference between the two biological classes of FLT3 inhibitors. This underscores the unmet need for prospective studies to compare the efficacies of type 1 and type 2 FLT3 inhibitors in treating patients with *FLT3*‐mutated AML. Also, with the availability of several FLT3 inhibitors, randomized trials are needed to dissect the choices regarding the class of FLT3 inhibitors to be used in the de novo and R/R AML setting.

## AUTHOR CONTRIBUTIONS

Mahesh Swaminathan was involved with study design, curating data, methodology, and writing—original draft. Mai M. Aly involved with study design, curating data, and methodology.

Abdul Moiz Khan was involved in curating data and editing manuscript. Bayan Al Share, Vikram Dhillon, Enxhi lalo and Harry Ramos were involved in curating data, and analysis. Katherine G. Akers involved in study design, extraction of data from scientific databases and creating PRISMA flow diagram. Seongho Kim involved with the study design and statistical analysis. Suresh Balasubramanian involved with study design, methodology, analysis, overall conduct, and manuscript editing.

## CONFLICT OF INTEREST

The authors declare they have no conflicts of interest.

## FUNDING INFORMATION

The authors received no specific funding for this work.

## ETHICS STATEMENT

None.

## Supporting information

FiguresS1‐S2ACKNOWLEDGEMENT
