# Supplementary material for: Efficacy analysis of different FLT3 inhibitors in patients with relapsed/refractory acute myeloid leukemia and high‐risk myelodysplastic syndrome
Source: EJHaem. 2022 Nov 21;4(1):165–73. doi: 10.1002/jha2.616 (PMC9928788; doi:10.1002/jha2.616)

**Supplemental File**

**Supplemental Figure 1**: Type 1 FLT3: inhibitors bind the FLT3 receptor in the active conformation, either near the activation loop or the ATP binding pocket, and are active against ITD and TKD mutations. Type 2 FLT3 inhibitors bind the FLT3 receptor in the inactive conformation in a region adjacent to the ATP-binding domain. As a result of this binding affinity, type 2 FLT3 inhibitors prevent activity of ITD mutations but do not target TKD mutations. FLT3, FMS-like tyrosine kinase; ITD, internal tandem duplication; JMD, juxtamembrane domain; TK, tyrosine kinase; TKD, tyrosine kinase domain.


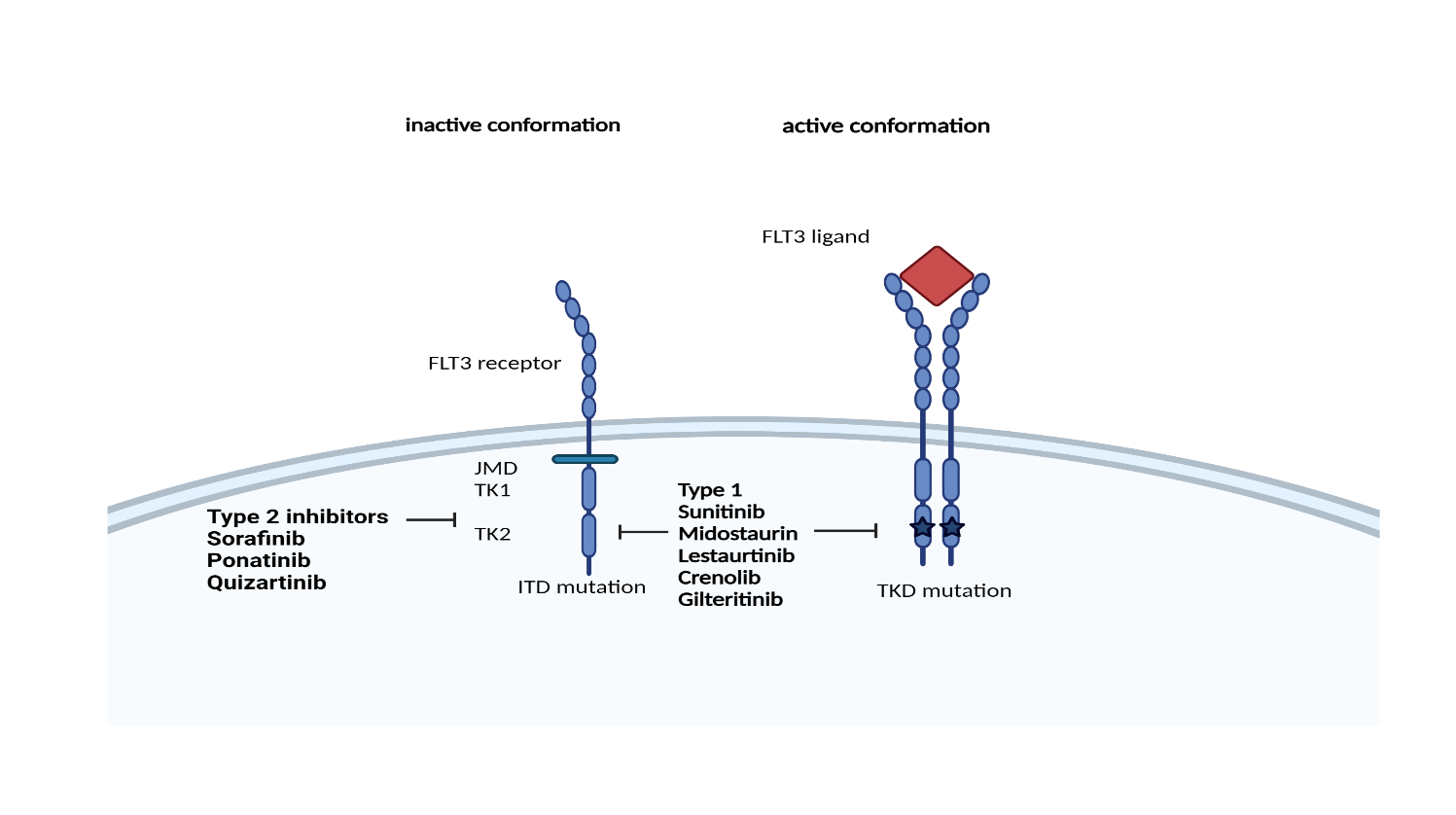


**Supplemental Figure 2**: Forest plots of the meta-analysis of overall response rate (ORR) for (a) Type 1 and (b) Type 2. Type 2 was adjusted for publication bias by the trim-and-fill method (left). Forest plot of the meta-analysis of complete response rate (CRc) after adjusting publication bias by the trim-and-fill method for (a) Type 1 and (b) Type 2 (right).


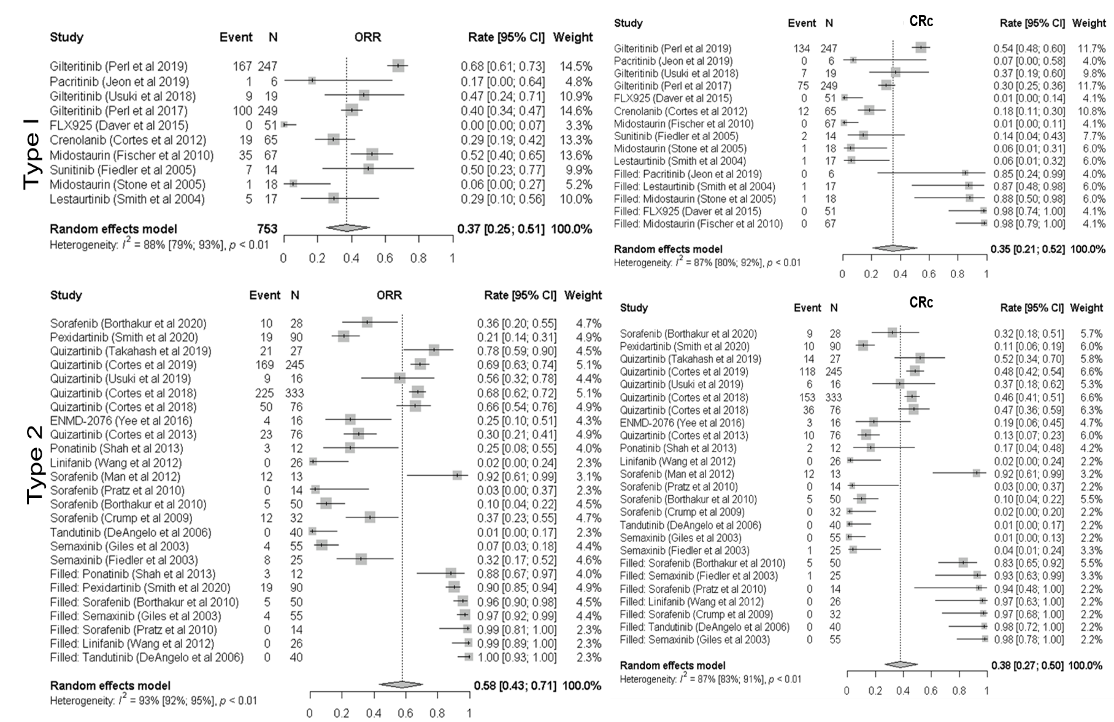

Supplement: Supplementary file 1 — FiguresS1‐S2ACKNOWLEDGEMENT [file JHA2-4-165-s001.docx]
